# Association of Cytomegalovirus End-Organ Disease with Stroke in People Living with HIV/AIDS: A Nationwide Population-Based Cohort Study

**DOI:** 10.1371/journal.pone.0151684

**Published:** 2016-03-17

**Authors:** Yung-Feng Yen, Ian Jen, Marcelo Chen, Pei-Hung Chuang, Yen-Ling Liu, Gerald B. Sharp, Yi-Ming Arthur Chen

**Affiliations:** 1 Section of Infectious Diseases, Taipei City Hospital, Taipei City Government, Taipei, Taiwan; 2 School of Medicine, National Yang-Ming University, Taipei, Taiwan; 3 Center for Infectious Disease and Cancer Research, Kaohsiung Medical University, Kaohsiung, Taiwan; 4 Department and Institute of Public Health, National Yang-Ming University, Taipei, Taiwan; 5 Department of Urology, Mackay Memorial Hospital, Taipei, Taiwan; 6 Department of Cosmetic Applications and Management, Mackay Junior College of Medicine, Nursing and Management, Taipei, Taiwan; 7 Center for prevention and treatment of occupational injury and diseases, Taipei Veterans General Hospital, Taipei, Taiwan; 8 Division of Clinical Toxicology and Occupational Medicine, Department of Medicine, Taipei Veterans General Hospital, Taipei, Taiwan; 9 Epidemiology Branch, Basic Sciences Program, Division of AIDS, National Institute of Allergy and Infectious Diseases, National Institutes of Health, Bethesda, Maryland, United States of America; 10 Department of Microbiology and Institute of Medical Research, College of Medicine, Kaohsiung Medical University, Kaohsiung, Taiwan; National Institute of Child Health and Human Development, UNITED STATES

## Abstract

**Objectives:**

Cytomegalovirus (CMV) infection might increase the risk of cardiovascular event. However, data on the link between incident stroke and co-infections of CMV and human immunodeficiency virus (HIV) are limited and inconsistent. This nationwide population-based cohort study analyzed the association of CMV end-organ disease and stroke among people living with HIV/AIDS (PLWHA).

**Methods:**

From January 1, 1998, this study identified adult HIV individuals with and without CMV end-organ disease in the Taiwan National Health Insurance Research Database. All patients were observed for incident stroke and were followed until December 31, 2012. Time-dependent analysis was used to evaluate associations of CMV end-organ disease with stroke.

**Results:**

Of the 22,581 PLWHA identified (439 with CMV end-organ disease and 22,142 without CMV end-organ disease), 228 (1.01%) had all-cause stroke during a mean follow-up period of 4.85 years, including 169 (0.75%) with ischemic stroke and 59 (0.26%) with hemorrhagic stroke. After adjusting for age, sex, comorbidities, opportunistic infections after HIV diagnosis, and antiretroviral treatment, CMV end-organ disease was found to be an independent risk factor for incident all-cause stroke (adjusted hazard ratio [AHR], 3.07; 95% confidence interval [CI], 1.70 to 5.55). When stroke type was considered, CMV end-organ disease was significantly positively associated with the risk of ischemic stroke (AHR, 3.14; 95% CI, 1.49 to 6.62) but not hemorrhagic stroke (AHR, 2.52; 95% CI, 0.64 to 9.91).

**Conclusions:**

This study suggested that CMV end-organ disease was an independent predictor of ischemic stroke among PLWHA.

## Introduction

Stroke remains a leading cause of mortality worldwide [1]. Stroke is a complicated disease and is influenced by genetic and environmental factors and their interactions [2]. Accumulating evidence indicates that inflammation is important in stroke development [3–5].

Human cytomegalovirus (CMV) is a ubiquitous DNA virus of the *Herpesviridae* family that replicates only in humans. CMV infection is usually asymptomatic in immunocompetent patients. However, CMV infection can cause serious diseases (e.g., retinitis) in people living with HIV/AIDS (PLWHA) [6]. Human CMV also inhibits Akt-mediated endothelial nitric oxide synthase activation, thereby resulting in endothelial dysfunction and exerting a proatherogenic effect [7]. In addition, CMV infection of vascular smooth muscle cells induces production of powerful proinflammatory cytokines (e.g., leukotriene B_4_), which accelerate atherosclerosis development [8, 9].

Despite accumulating evidence suggesting that active CMV replication in vascular cells leads to atherosclerosis and stenosis [7, 8, 10], few studies have investigated the association between CMV infection and stroke. A prior case study reported that severe CMV infection caused central nervous system vasculitis and resulted in hemiplegia [11]. A recent observational study showed that CMV seropositivity was associated with increased risk of stroke [12], but another study found no significant association [13].

Stroke management and prevention should include identification and prevention of specific stroke risk factors, particularly in high-risk populations. Therefore, we conducted a nationwide population-based cohort study of the association between CMV end-organ disease and stroke among PLWHA in Taiwan during the period from 1998 through 2012.

## Methods

### Background information

Taiwan launched its single-payer National Health Insurance program in 1995 [14]. Our nationwide cohort study analyzed patient data obtained from the National Health Insurance Research Database (NHIRD), which contains health care data from more than 99% of the population in Taiwan [15]. The NHIRD is a large-scale computerized database derived from the system used by the Bureau of National Health Insurance (NHI) and is provided to scientists for research purposes. Patient identification codes in the NHIRD are scrambled and de-identified before being accessed by the researchers. With approval from the National Health Research Institutes, the NHIRD was accessed at the Collaboration Center of Health Information Application (CCHIA), Department of Health, Executive Yuan, Taiwan. In the NHIRD, the accuracy of diagnoses of major diseases, such as diabetes mellitus and cerebrovascular disease, has been well validated [16, 17]. This study was approved by the institutional review board of Kaohsiung Medical University.

### Study subjects

In this cohort study, we selected adult subjects aged 15 years or older who were newly diagnosed with HIV between January 1, 1998 and December 31, 2012. A new case of HIV was defined as a patient for whom there was (1) a record of a relevant International Classification of Diseases, Ninth Revision, Clinical Modification (ICD-9-CM) code (042 to 044, 7958, or V08) in an inpatient setting or three or more outpatient visits, and (2) a record of an examination for viral load (order codes: 14074B) or CD4 count (order codes: 26017A1, 14074B, 12071A, 12071B, 12073A, 12073B, 12074A, 12074B) [18]. Patients were excluded if they received a diagnosis of stroke (ICD-9-CM codes 430 to 437) or CMV infection (ICD-9-CM code 078.5) before an HIV diagnosis. All study subjects were followed until the study endpoints of hospitalization for stroke unclassified (ICD-9CM codes 430–437), hemorrhagic stroke (ICD-9-CM codes 430–432), or ischemic stroke (ICD-9-CM codes 433 to 437) [19] or until the study endpoint of death as recorded in Taiwan’s national death certificate database or until December 31, 2012, the end date of the study.

### Independent and potential confounding variables

The study’s main explanatory variable was CMV end-organ disease after HIV diagnosis. CMV end-organ disease was defined as ICD-9-CM code 078.5 plus a prescription for an anti-CMV drug (e.g., ganciclovir or valganciclovir). Confounders controlled in the analysis included sociodemographics, comorbidities, history of opportunistic infection (OI) after HIV diagnosis, and treatment with highly active antiretroviral therapy (HAART). Sociodemographic variables included income level and urban or rural residence. Income level was calculated as the average monthly income, classified as low (≤19,200 New Taiwan Dollars [NTD]), intermediate (19,201 NTD to 39,999 NTD), or high (≥40,000 NTD).

The comorbidities analyzed included diabetes (ICD-9 code 250), chronic kidney disease (ICD-9 codes 580–587), hypertension (ICD-9 codes 401–405), coronary heart disease (ICD-9 codes 410–414), cancer (ICD-9 codes 140–208), and dyslipidemia (ICD-9 code 272). OIs after HIV diagnosis included *Mycobacterium tuberculosis* infection (ICD-9 codes 011–018), disseminated *Mycobacterium avium* complex infection (ICD-9 code 0312), *Pneumocystis jirovecii* pneumonia (ICD-9 code 1363), cryptococcal meningitis (ICD-9 code 3210), *Penicillium marneffei* infection (ICD-9 code 1179), toxoplasma encephalitis (ICD-9 code 130), candidiasis (ICD-9 code 112), and herpes zoster (ICD-9 code 053). Subjects were classified as having a comorbidity or OI only if the condition occurred in an inpatient setting or was recorded in three or more outpatient visits [20].

### Statistical analysis

Comparisons between groups were made using the two-sample t test, and the Pearson χ^2^ test was used to analyse categorical data. The incidence of ischemic, hemorrhagic, and all-cause stroke per 1,000 person-years and the relative hazards (RH) of stroke were calculated for HIV patients with and without CMV end-organ disease, the latter being estimated from Cox proportional-hazards models.

To identify independent risk factors for incident stroke, a time-dependent Cox proportional-hazards model was used to calculate hazard ratios (HRs) and 95% confidence intervals (CIs), adjusting for age, sex, comorbidities, OIs, and HAART use. In these models, HAART use, CMV end-organ disease, and other OIs were regarded as time-dependent covariables [11], whereas other confounders including age, sex, and history of comorbidities, which were collected at baseline, were considered as fixed covariates. Adjusted HRs (AHRs) with 95% CIs were calculated to indicate the strength and direction of associations.

To examine the robustness of the main findings, we conducted sensitivity analyses after stratifying study subjects by age, sex, comorbidities, OIs, and HAART treatment. All statistical analyses were performed using the SAS 9.4 software package (SAS Institute, Cary, NC).

## Results

### Patient selection

**Fig 1:**
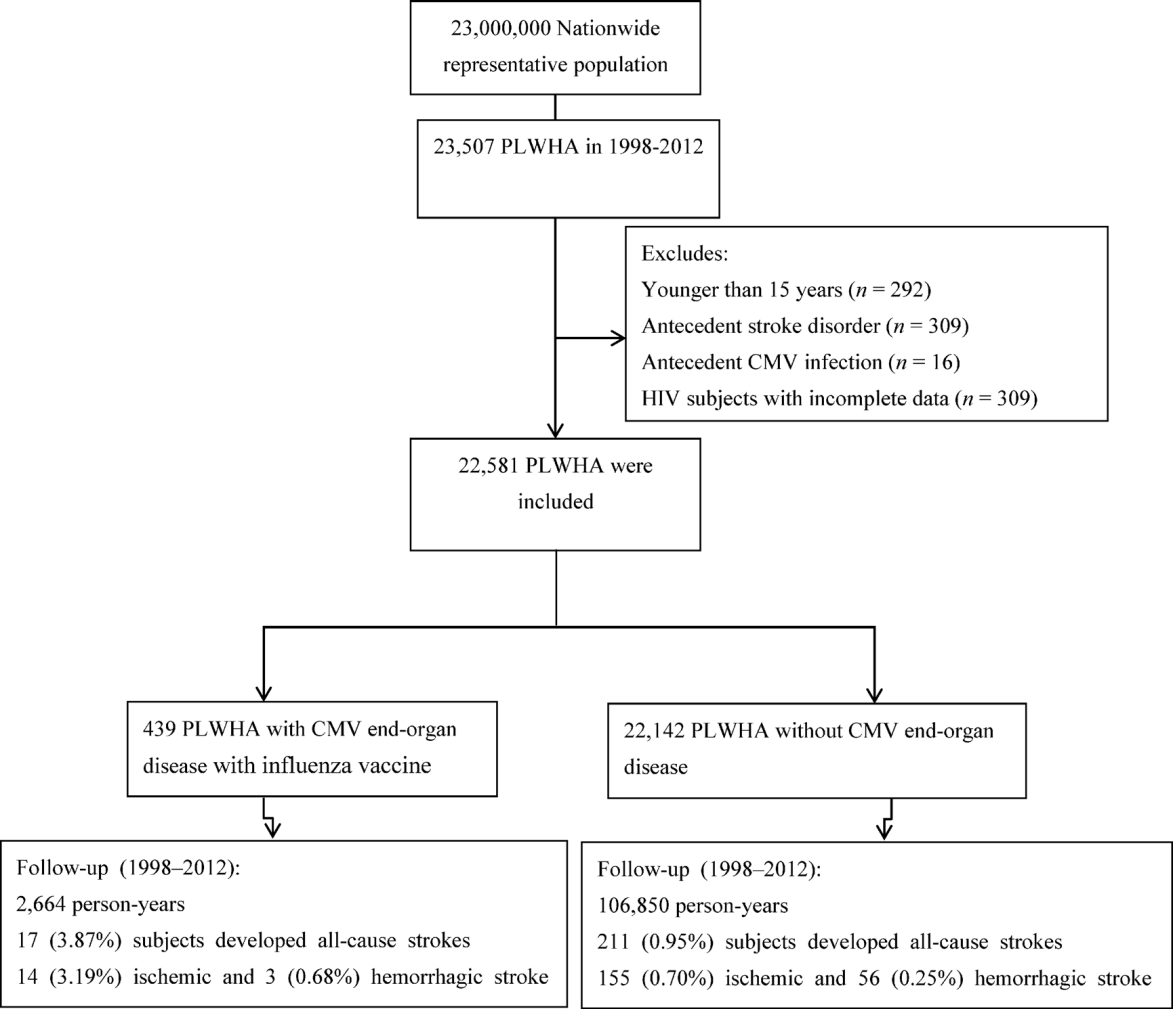
Study flow diagram. PLWHA, people living with HIV/AIDS; CMV, cytomegalovirus.

We identified 23,507 individuals who were diagnosed with HIV from January 1, 1998 through December 31, 2012. After excluding those younger than 15 years (*n* = 292) and those with antecedent stroke disorder (*n* = 309), antecedent CMV infection (*n* = 16), or incomplete data (*n* = 309), 22,581 PLWHA remained for analysis (Fig 1). The overall mean (SD) age was 34.2 (10.8) years, and 91.1% of the subjects were male. During the follow-up period, 228 (1.01%) PLWHA had new onset of stroke, including 169 (0.75%) ischemic strokes and 59 (0.26%) hemorrhagic strokes. Among the 439 PLWHA with CMV end-organ disease, there were 17 (3.87%) all-cause strokes, including 14 (3.19%) ischemic strokes and 3 (0.68%) hemorrhagic strokes. Among the 22,142 PLWHA without CMV end-organ disease, there were 211 (0.95%) all-cause strokes, including 155 (0.70%) ischemic strokes and 56 (0.25%) hemorrhagic strokes.

### Demographic characteristics and comorbidities

Table 1 shows the demographic characteristics and comorbidities of PLWHA with and without CMV end-organ disease. There was no significant difference in age, sex, or comorbidities between these groups. As compared with PLWHA without CMV end-organ disease, PLWHA with CMV end-organ disease were more likely to have other OIs during the study follow-up period. In addition, PLWHA with CMV end-organ disease were more likely to have received HAART than those without CMV end-organ disease.

**Table 1:** Characteristics of PLWHA with and without CMV End-organ Disease.

Characteristic	No. (%) of subjects*		P
	With cytomegalovirus end-organ disease, n = 439	Without cytomegalovirus end-organ disease, n = 22142	
Demographics			
Age, yr			
Mean ± SD	34.2 ± 10.6	34.2 ± 10.9	0.87
15–49	401 (91.34)	20199 (91.22)	0.96
≥50	38 (8.66)	1943 (8.78)	
Sex			
Male	411 (93.62)	20164 (91.07)	0.062
Female	28 (6.38)	1978 (8.93)	
Income level			
Low	265 (60.36)	13023 (58.82)	0.663
Intermediate	122 (27.79)	6594 (29.78)	
High	52 (11.85)	2525 (11.40)	
Urbanization			
Rural	51 (11.62)	4246 (19.18)	<0.001
Urban	388 (88.38)	17896 (80.82)	
Comorbidities			
Diabetes	7 (1.59)	364 (1.64)	0.936
CKD	4 (0.91)	121 (0.55)	0.308
HTN	10 (2.28)	518 (2.34)	0.933
CHD	2 (0.46)	155 (0.70)	0.542
Dyslipidemia	4 (0.91)	190 (0.86)	0.905
Cancer	14 (3.19)	495 (2.24)	0.183
Opportunistic infections after HIV diagnosis			
TB infection	150 (34.17)	1269 (5.73)	< .001
Disseminated Mycobacterium avium complex infection	36 (8.20)	93 (0.42)	< .001
Pneumocystis jirovecii pneumonia	220 (50.11)	1131 (5.11)	< .001
Cryptococcal meningitis	22 (5.01)	160 (0.72)	< .001
Candidiasis	186 (42.37)	1473 (6.65)	< .001
Penicillium marneffei infection	17 (3.87)	134 (0.61)	< .001
Toxoplasma encephalitis	10 (2.28)	53 (0.24)	< .001
Herpes zoster	83 (18.91)	1559 (7.04)	< .001
HAART			
No	12 (2.73)	7623 (34.43)	<0.001
Yes	427 (97.27)	14519 (65.57)	
Follow-up years, mean (SD)	6.07 (3.79)	4.83 (3.69)	< .001

PLWHA, people living with HIV/AIDS; SD, standard deviation; HIV, human immunodeficiency virus; CMV, cytomegalovirus; CKD, chronic kidney disease; HTN, hypertension; CHD, coronary heart disease; TB, tuberculosis; HAART, highly active antiretroviral therapy.

*Unless otherwise stated.

### Incidence rate of stroke

The incidence rate of all-cause stroke per 1,000 person-years (py) was 6.38 for PLWHA with CMV end-organ disease and 1.97 for PLWHA without CMV end-organ disease (P <0.001) (Table 1), which was primarily attributable to a significantly higher incidence of ischemic stroke for PLWHA with CMV end-organ disease (incidence rate per 1,000 py: 5.26 vs. 1.45; P <0.001). There was no significant difference in the incidence of hemorrhagic stroke for PLWHA with and without CMV end-organ disease (incidence rates per 1000 py: 1.13 and 0.55, respectively; p = 0.181). As compared with PLWHA without CMV end-organ disease, the RHs of incident ischemic, hemorrhagic, and all-cause stroke among PLWHA with CMV end-organ disease were 3.28 (95% CI, 1.85 to 5.80), 2.15 (95% CI, 0.67 to 6.89), and 3.00 (95% CI, 1.80 to 4.99), respectively.

The mean (SD) time to onset of incident all-cause stroke was 4.1 (3.4) and 4.6 (3.7) years from HIV diagnosis in stroke patients with and without CMV end-organ disease, respectively (P <0.001). Among stroke patients with CMV end-organ disease, the mean (SD) time to onset of incident all-cause stroke was 1.2 (1.2) years from CMV end-organ disease. Also, among 17 stroke patients with CMV end-organ disease, 53.0% (9) occurred within one year of CMV end-organ disease; 23.5% (4) occurred between one and two years of CMV end-organ disease; and 23.5% (4) occurred after two years of CMV end-organ disease.

### Association of CMV end-organ disease with incident all-cause stroke

A time-dependent Cox proportional-hazards model was used to identify independent risk factors for all-cause stroke. After adjusting for age, sex, comorbidities, OIs, and HAART, CMV end-organ disease was associated with a significantly increased risk of incident all-cause stroke (AHR, 3.07; 95% CI, 1.70 to 5.55) (Table 2). Other risk factors for incident all-cause stroke included age ≥50 years (AHR, 6.37; 95% CI, 4.76 to 8.54), hypertension (AHR, 2.11; 95% CI, 1.29 to 3.44), tuberculosis (TB) infection (AHR 1.51; 95% CI, 1.02 to 2.24), and cryptococcal meningitis (AHR, 5.24; 95% CI, 2.53 to 10.82). As compared with PLWHA with low income, those with intermediate income had a lower risk of incident all-cause stroke (AHR 0.59; 95% CI, 0.42 to 0.83). Moreover, PLWHA receiving HAART had a lower risk of incident all-cause stroke (AHR, 0.48; 95% CI, 0.35 to 0.64) than those who did not receive HAART.

**Table 2:** Univariate and Multivariate Analyses of the Association of CMV End-organ Disease and All-cause Stroke among PLWHA.

Characteristic	Number of patients	All-cause Stroke	Univariate analysis	Multivariates analysis
		n (%)	HR (95% CI)	AHR (95% CI)
Cytomegalovirus end-organ disease				
No	22142	211 (0.95)	1	1
Yes	439	17 (3.87)	3.00 (1.80–4.99)***	3.07 (1.70–5.55)***
Demographics				
Age, yr				
15–49	20600	134 (0.65)	1	1
≥50	1981	94 (4.75)	7.69 (5.90–10.01)***	6.37 (4.76–8.54)***
Sex				
Male	20575	189 (0.94)	1	1
Female	2006	37 (1.88)	1.63 (1.15–2.32)**	0.95 (0.65–1.38)
Income level				
Low	13288	163 (1.23)	1	1
Intermediate	6716	41 (0.61)	0.55 (0.39–0.77)***	0.59 (0.42–0.83)**
High	2577	24 (0.93)	0.73 (0.48–1.12)	0.77 (0.50–1.18)
Urbanization				
Rural	4297	52 (1.21)	1	1
Urban	18284	176 (0.99)	0.66 (0.49–0.91)**	0.84 (0.61–1.17)
Comorbidity				
Diabetes				
No	22210	216 (0.99)	1	1
Yes	371	12 (3.23)	4.44 (2.48–7.95)***	1.16 (0.60–2.24)
CKD				
No	22456	225 (1.00)	1	1
Yes	125	3 (2.40)	3.27 (1.05–10.22)*	1.18 (0.37–3.82)
HTN				
No	22053	202 (0.92)	1	1
Yes	528	26 (4.92)	6.99 (4.64–10.53)***	2.11 (1.29–3.44)**
CHD				
No	22424	224 (1.00)	1	1
Yes	157	4 (2.55)	2.89 (1.07–7.76)*	0.43 (0.15–1.22)
Dyslipidemia				
No	22387	221 (0.99)	1	1
Yes	194	7 (3.61)	4.79 (2.26–10.16)***	1.31 (0.57–3.02)
Cancer				
No	22072	218 (0.99)	1	1
Yes	509	10 (1.96)	2.58 (1.37–4.86)**	1.31 (0.68–2.52)
Opportunistic infections after HIV diagnosis				
TB infection				
No	21162	184 (0.87)	1	1
Yes	1419	44 (3.10)	1.75 (1.21–2.51)**	1.51 (1.02–2.24)*
Disseminated Mycobacterium avium complex infection				
No	22452	224 (1.00)	1	1
Yes	129	4 (3.10)	2.84 (1.06–7.62)*	1.94 (0.68–5.53)
Pneumocystis jirovecii pneumonia				
No	21230	209 (0.98)	1	1
Yes	1351	19 (1.41)	1.19 (0.74–1.90)	1.03 (0.60–1.77)
Cryptococcal meningitis				
No	22399	220 (0.98)	1	1
Yes	182	8 (4.40)	4.26 (2.10–8.62)***	5.24 (2.53–10.82)***
Candidiasis				
No	20922	203 (0.97)	1	1
Yes	1659	25 (1.51)	1.14 (0.75–1.72)	0.98 (0.61–1.56)
Penicillium marneffei infection				
No	22430	223 (0.99)	1	1
Yes	151	5 (3.31)	2.22 (0.91–5.38)	1.75 (0.70–4.36)
Toxoplasma encephalitis				
No	22518	226 (1.00)	1	1
Yes	63	2 (3.17)	2.73 (0.68–10.98)	2.25 (0.55–9.25)
Herpes zoster				
No	20939	207 (0.99)	1	1
Yes	1642	21 (1.28)	0.87 (0.55–1.36)	0.78 (0.49–1.23)
HAART				
No	7635	81 (1.06)	1	1
Yes	14946	147 (0.98)	0.44 (0.34–0.58)***	0.48 (0.35–0.64)***

* < .05

** < .01

*** < .001

PLWHA, people living with HIV/AIDS; AHR, adjusted hazard ratio; CI, confidence interval; HIV, human immunodeficiency virus; CMV, cytomegalovirus; CKD, chronic kidney disease; HTN, hypertension; CHD, coronary heart disease; TB, tuberculosis; HAART, highly active antiretroviral therapy.

### Association of CMV end-organ disease with incident ischemic and hemorrhagic stroke

Table 3 shows the time-dependent multinomial Cox regression for factors associated with ischemic and hemorrhagic stroke among PLWHA. CMV end-organ disease significantly increased the risk of incident ischemic stroke (AHR, 3.14; 95% CI, 1.49 to 6.62) but not incident hemorrhagic stroke (AHR, 2.52; 95% CI, 0.64 to 9.91).

**Table 3:** Time-dependent Multinomial Regression Analysis of the Association of CMV End-organ Disease with Ischemic and Hemorrhagic Stroke among PLWHA.

Variables	Ischemic stroke	Hemorrhagic stroke
	AHR (95% CI)	AHR (95% CI)
Cytomegalovirus end-organ disease	3.14 (1.49–6.62)**	2.52 (0.64–9.91)
Demographics		
Age, yr		
15–49	1	1
≥50	7.22 (5.14–10.13)***	4.10 (2.02–8.32)***
Sex		
Male	1	1
Female	1.18 (0.78–1.79)	0.46 (0.17–1.24)
Income level		
Low	1	1
Intermediate	1.04 (0.65–1.67)	3.00 (0.90–9.97)
High	0.70 (0.40–1.21)	1.20 (0.32–4.55)
Urbanization		
Rural	1	1
Urban	0.86 (0.59–1.26)	0.79 (0.45–1.40)
Comorbidity		
Diabetes	1.61 (0.73–3.58)	0.35 (0.04–3.26)
CKD	-	5.77 (1.28–26.09)*
HTN	1.57 (0.81–3.02)	3.86 (1.44–10.40)**
CHD	0.58 (0.19–1.83)	-
Dyslipidemia	1.43 (0.52–3.95)	0.75 (0.10–5.97)
Cancer	1.04 (0.43–2.52)	2.05 (0.60–7.04)
Opportunistic infections after HIV diagnosis		
TB infection	1.56 (0.98–2.49)	1.36 (0.60–3.11)
Disseminated Mycobacterium avium complex infection	2.49 (0.78–7.96)	-
Pneumocystis jirovecii pneumonia	1.16 (0.60–2.22)	0.69 (0.18–2.66)
Cryptococcal meningitis	5.86 (2.67–12.85)***	2.71 (0.41–18.00)
Candidiasis	0.94 (0.52–1.72)	1.19 (0.45–3.16)
Penicillium marneffei infection	1.27 (0.38–4.30)	3.31 (0.68–16.05)
Toxoplasma encephalitis	2.90 (0.61–13.68)	-
Herpes zoster	0.73 (0.42–1.27)	1.01 (0.44–2.33)
HAART		
No	1	1
Yes	0.52 (0.37–0.74)***	0.41 (0.23–0.72)**

* < .05

** < .01

*** < .001

PLWHA, people living with HIV/AIDS; AHR, adjusted hazard ratio; CI, confidence interval; HIV, human immunodeficiency virus; CMV, cytomegalovirus; CKD, chronic kidney disease; HTN, hypertension; CHD, coronary heart disease; TB, tuberculosis; HAART, highly active antiretroviral therapy.

### Sensitivity analysis of the association between CMV end-organ disease and stroke

**Fig 2:**
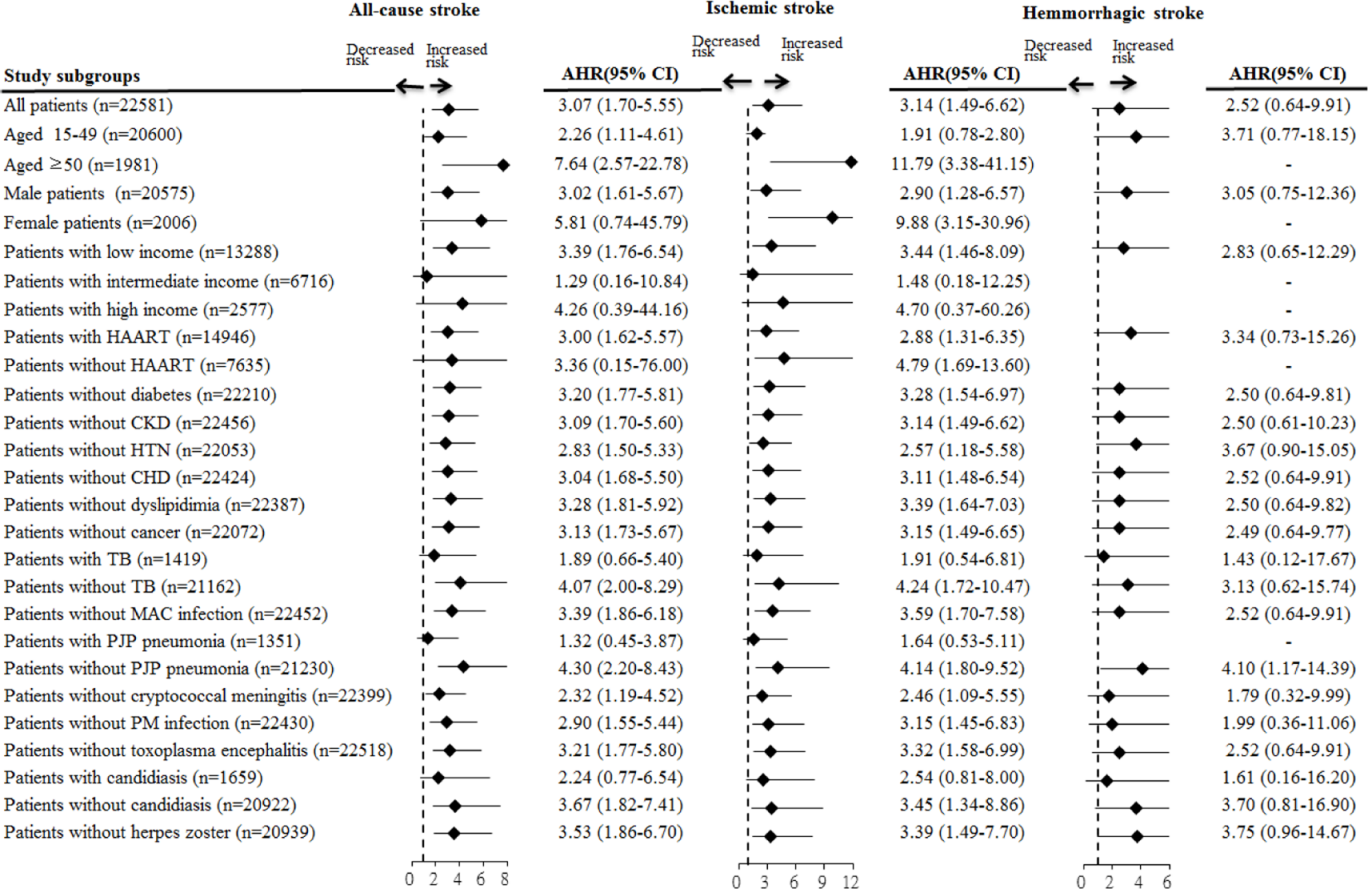
Sensitivity analysis of the association between CMV end-organ disease and stroke in PLWHA subgroups, after adjustment for patient demographics, comorbidities, and antiretroviral treatment. **Values greater than 1.0 indicate increased risk.** AHR, adjusted hazard ratio; CMV, cytomegalovirus; PLWHA, people living with HIV/AIDS; HAART, highly active antiretroviral therapy; CHD, coronary heart disease; MAC, Mycobacterium avium complex; PJP, Pneumocystis jirovecii pneumonia; PM, Penicillium marneffei.

Fig 2 shows the results of sensitivity analysis of the association between CMV end-organ disease and stroke after adjusting for patient demographics, comorbidities, OIs, and HAART. CMV end-organ disease was significantly associated with higher risk of all-cause stroke in all PLWHA subgroups except female patients, those not receiving HAART, those with intermediate or high income, and those with TB infection, *P*. *jirovecii* pneumonia, or candidiasis. In addition, CMV end-organ disease significantly increased the risk of ischemic stroke in all PLWHA subgroups except patients aged 15 to 49 years, those with intermediate or high income, and those with TB infection, *P*. *jirovecii* pneumonia, or candidiasis. CMV end-organ disease was not associated with a higher risk of hemorrhagic stroke in any PLWHA subgroup.

## Discussion

In this large cohort study of 22,581 Taiwanese PLWHA, the overall incidence rate of all-cause stroke was 2.08 per 1,000 py. Controlling for potential confounders, the risk of incident all-cause stroke was higher among PLWHA with CMV end-organ disease than among those without CMV end-organ disease. When type of stroke was considered, CMV end-organ disease significantly increased the risk of ischemic stroke but not hemorrhagic stroke.

This study found that the incidence rates of all-cause, ischemic, and hemorrhagic strokes were 2.08, 1.54, and 0.54 per 1,000 py among PLWHA, respectively. In a comparison with other countries, the rate of incident all-cause stroke among PLWHA in Taiwan was lower than that in Danish HIV cohort (2.68 per 1000 PY) [21]. Also, the rate of incident ischemic stroke among PLWHA in Taiwan was similar to that in Swiss HIV cohort (1.73 per 1000 PY) [22], but lower than that among PLWHA in U.S. (5.27 per 1000 PY) [22].An association between CMV disease and vasculopathy was reported in previous studies [23]. However, findings from studies of the link between CMV disease and incident stroke are limited and inconsistent [11–13]. A case study reported that CMV end-organ disease in an HIV-infected patient induced central nervous system vasculitis, which resulted in multiple small-vessel cerebral infarctions [11]. The magnetic resonance imaging findings showed interval evolution of infarctions while the patient received valganciclovir treatment [11]. A recent observational study found that the proportion of patients with CMV seropositivity was significantly higher among stroke patients than among matched non-stroke patients [12]. However, a recent Italian Cohort Naive Antiretrovirals (ICONA) study showed that HIV patients who were CMV-seropositive at baseline did not have a significantly higher risk of cerebrovascular diseases than those who were CMV-seronegative [13]. Although the ICONA study was the first cohort study to evaluate the link between CMV infection and stroke, the association of CMV infection and stroke might have been underestimated in that study because many of the CMV-seropositive patients could have had asymptomatic CMV infection [13]. A prior study showed that asymptomatic CMV infection was not associated with atherosclerosis development [24]. The present study followed 22,050 HIV patients and evaluated the association of CMV end-organ disease with stroke. Time-dependent Cox regression showed that CMV end-organ disease significantly increased the risk of all-cause stroke. When type of stroke was considered, CMV end-organ disease significantly increased the risk of ischemic stroke but not hemorrhagic stroke. These findings suggest that CMV end-organ disease was an independent predictor of ischemic stroke among PLWHA.

CMV-induced inflammation, vasculopathy, and atherosclerosis may have increased the risk of incident stroke among PLWHA with CMV end-organ disease in this study. CMV end-organ disease of smooth muscle cells can cause vascular cell inflammation and proliferation through generation of reactive oxygen species and activation of nuclear factor kB [9, 25]. CMV end-organ disease of smooth muscle cells can also initiate proinflammatory gene transcription (e.g., 5-lipoxygenase expression), while 5-lipoxgenase induces synthesis of leukotriene B_4_, a powerful proinflammatory cytokine, and may contribute to atherosclerosis and stenosis [8]. Also, CMV end-organ disease of vascular cells caused the stimulation of fractalkine-CX3CR1 interaction, which resulted in the endothelial damage [26]. In addition, human CMV inhibits Akt-mediated endothelial nitric oxide synthase activation, which leads to endothelial dysfunction and exerts a proatherogenic effect [7]. Taken together, these mechanisms indicate that active CMV replication in vascular endothelial cells may create an atherosclerotic environment that results in restriction of blood flow and promotion of ischemic stroke.

This study is the largest cohort study of the association between CMV end-organ disease and subsequent stroke development. Our research design, which included unbiased subject selection and strict criteria for CMV diagnosis, increased the validity of the findings. In addition, this nationwide population-based study traced all PLWHA, and referral bias was minimized because all medical care was covered by the Taiwan National Health Insurance. Moreover, the large sample size was powered to detect even very small differences between PLWHA with and without CMV end-organ disease. Additionally, the timing of CMV end-organ disease was ascertained in all patients, and CMV end-organ disease was regarded as a time-dependent variable in the analysis. Longitudinal studies that do not account for changes in exposure during the study period do not yield precise estimates of the effect of the exposure on outcomes [27].

The present study has some limitations. First, some potential risk factors, including smoking and obesity, could not be ascertained. However, some smoking-related comorbidities (e.g., hypertension and coronary heart disease) were included in the analysis. Second, diagnoses of CMV end-organ disease and stroke that rely on administrative claims data recorded by physicians or hospitals may be less accurate than diagnoses made in a prospective clinical setting, but there is no reason to suspect that the validity of claims data would differ with a patient’s CMV status. CMV end-organ disease in this study was defined strictly according to the relevant ICD-9 codes and was confirmed by records of treatment with anti-CMV drugs, thereby maximizing diagnostic validity. Furthermore, a stroke event was defined as a patient hospitalized for stroke. A previous study confirmed that the accuracy of the Taiwan NHIRD in recording stroke diagnoses was high (98%) [16]. Third, CD4 counts and viral loads—the index of advanced-stage of HIV infection—were unavailable in our database. However, our study used the opportunistic infections as the proxy for advanced-stage of HIV infection. All opportunistic infections among PLWHA were included in the multivariate analysis. Finally, this study was limited to the evaluation of the association between CMV end-organ disease and incident stroke among PLWHA. Although this study revealed an association between CMV end-organ disease and stroke, the generalizability of our findings to other non-HIV subgroups requires confirmation.

## Conclusion

This nationwide, long-term cohort study revealed a link between CMV end-organ disease and stroke among PLWHA. CMV end-organ disease was associated with a higher risk of incident all-cause stroke. When type of stroke was considered, CMV iend-organ disease was significantly associated with an increased risk of ischemic stroke but not hemorrhagic stroke. These findings suggest that CMV end-organ disease was an independent predictor of ischemic stroke among PLWHA.
